# SOX2 promoter hypermethylation in non-smoking Taiwanese adults residing in air pollution areas

**DOI:** 10.1186/s13148-019-0647-8

**Published:** 2019-03-12

**Authors:** Disline Manli Tantoh, Ming-Fang Wu, Chien-Chang Ho, Chia-Chi Lung, Kuan-Jung Lee, Oswald Ndi Nfor, Yi-Chia Liaw, Shu-Yi Hsu, Pei-Hsin Chen, Chin Lin, Hou-Wei Chu, Yi-Ching Liaw, Yung-Po Liaw

**Affiliations:** 1 Department of Public Health and Institute of Public Health, Chung Shan Medical University, No. 110 Sec. 1 Jianguo N. Road, Taichung City, 40201 Taiwan; 2 School of Medicine, Chung Shan Medical University, Taichung City, Taiwan; 3 Department of Physical Education, Fu-Jen Catholic University, New Taipei City, Taiwan; 4 Department of Medical Education, Taipei Veterans General Hospital, Taipei, Taiwan; 5 Graduate Institute of Life Sciences, National Defense Medical Center, Taipei, Taiwan; 6 Institute of Biomedical Sciences, Academia Sinica, Taipei, Taiwan; 7 Institute of Clinical Medicine, National Yang-Ming University, Taipei, Taiwan; 8 Department of Family and Community Medicine, Chung Shan Medical University Hospital, Taichung City, Taiwan

**Keywords:** SOX2, Hypermethylation, Non-smoking, Air pollution, Biomarker, Taiwan Biobank

## Abstract

**Background:**

Both SOX2 promoter methylation and air pollution have been associated with lung cancer risk. However, little has been done to assess SOX2 promoter methylation in individuals living in air pollution areas. The aim of this study was to investigate SOX2 promoter methylation in non-smoking Taiwanese adults living in areas with different levels of air pollution especially particulate matter with diameter < 2.5 μm (PM_2.5_).

**Methods:**

A total of 1142 individuals aged 30–70 years were recruited. Data on SOX2 methylation, residence, age, and exposure to second-hand smoke (SHS) among others were extracted from the Taiwan Biobank dataset (2008–2015). After excluding former and current smokers, alongside those with incomplete information, a total of 461 non-smokers comprising 176 men and 285 women were included in the study. Participants’ residences were grouped under northern and central/southern areas because air pollution (PM_2.5_) is lower in northern compared to central and southern areas.

**Results:**

The methylation levels in men (0.16310 ± 0.01230) and women (0.15740 ± 0.01240) were significantly different (*P* < .0001). In both sexes, the SOX2 promoter region was shown to be significantly hypermethylated in central and southern areas compared with the northern areas. The regression coefficient (*β*) was 0.00331 (*P* = 0.0257) in men and 0.00514 (*P* < .0001) in women.

**Conclusion:**

SOX2 was significantly hypermethylated in both men and women residing in central and southern areas. The consistency in the results for both sexes shows that SOX2 promoter methylation could serve as a potential biomarker for industrial air pollution exposure. Moreover, it might reflect predisposition to cancer. Hence, healthy non-smokers at precancerous stages who have not been clinically diagnosed could be identified.

**Electronic supplementary material:**

The online version of this article (10.1186/s13148-019-0647-8) contains supplementary material, which is available to authorized users.

## Background

SOX2, also known as sex-determining region Y (SRY)-box 2, is an oncogenic transcription factor located on chromosome 3. It maintains pluripotency or self-renewal in embryonic stem cells and has been associated with several types of cancer [1–3]. In cancers, SOX2 promoter hypermethylation has been shown to be in constant correlation with an unfavorable clinical course and poor prognosis. For example, in esophageal squamous cell carcinoma, SOX2 promoter hypermethylation seemed to have caused SOX2 silencing and the loss of SOX2 expression was associated with poor prognostic outcome [3]. In lung cancer particularly, the overexpression of SOX2 has been associated with better prognosis and survival [2, 4, 5].

Lung cancer is one of the leading causes of global cancer mortality [6, 7]. In Taiwan, it was ranked the second incident cancer in 2014 and the top cause of mortality in 2016 [8]. Histologically, it is classified into small cell lung cancer (SCLC) and non-small cell lung cancer (NSCLC) [7]. SCLC and NSCLC respectively constitute about 15 and 85% of lung cancer [7]. SCLC is more common in former and current smokers even though certain uncommon cases have been reported in never-smokers [2, 6, 9–11]. NSCLC traditionally occurs in non-smokers and comprises three subtypes: adenocarcinoma (AC), squamous cell carcinoma (SCC), and large cell carcinoma (LCC) [7].

Several environmental, lifestyle, and genetic factors have been associated with lung cancer [6, 7, 11–14]. One of such factors is air pollution resulting from increased industrialization and urbanization [14–17]. Due to industrialization, PM_2.5_ pollution is lower in the northern regions compared to the central and southern regions of Taiwan [16, 18–20]. The carcinogenicity of air pollutants has not been fully explained. However, it is thought that markers like DNA methylation, mutation, and polymorphism could provide some insights into the mechanisms underlying this carcinogenicity [17].

SOX2 is believed to be a potential molecular marker for lung cancer [2]. In non-smokers exposed to air pollution, SOX2 promoter methylation might help in the early prediction of lung cancer risk. However, research has not been extensively conducted as far as this domain is concerned. Therefore, this study was conducted to investigate the association between SOX2 methylation in non-smoking Taiwanese adults residing in areas with different levels of air pollution especially PM_2.5_.

## Results

The basic characteristics of the participants are shown in Table 1. The methylation levels in men (0.16310 ± 0.01230) and women (0.15740 ± 0.01240) were significantly different (*P* < .0001). The mean (± SD) concentrations of PM_2.5_ (μg/m^3^) from 2006 to 2011 in the northern and central/southern areas are shown in Table 2. PM_2.5_ concentrations in central/southern regions were higher compared to the northern regions, irrespective of the year. After adjusting for age, exposure to second-hand smoke (SHS), exercise, drinking, body fat, BMI, waist-hip ratio (WHR), asthma, and emphysema, SOX2 promoter methylation was significantly hypermethylated in both men and women residing in central and southern areas compared with the northern areas (Tables 3 and 4). In men residing in the central/southern areas, the regression coefficient (*β*) was 0.00331; *P* = 0.0257 (Table 3). That is, the methylation level in male participants residing in central/southern areas was higher than in those residing in the northern areas and the difference was 0.00331. Similarly, in women residing in the central/southern areas, the regression coefficient (*β*) was 0.00514; *P* < 0.0001 (Table 4). That is, the methylation level in female participants residing in central/southern areas was higher than in those residing in the northern areas and the difference was 0.00514. Furthermore, the mean PM_2.5_ level (2006–2011) was significantly associated with higher SOX2 promoter methylation levels: *β* = 0.00042; *P* < 0.0001 (Table 5). That is, a unit increase in PM_2.5_ levels was associated with 0.00042 (*P* < 0.0001) higher methylation levels. Analysis of CpG sites both in and outside the SOX2 promoter region also showed significant hypermethylation in participants residing in central/southern compared with the northern areas (Additional file 1: Tables S1-S3). In addition, analysis of the KRAS gene promoter CpG sites also showed significant hypermethylation in participants residing in the central and southern compared with the northern areas (Additional file 1: Table S4).

**Table 1:** Basic characteristics of the study participants by sex

Variable	Men (*n* = 176)	Women (*n* = 285)	*P* value
SOX2 promoter beta value	0.1631 ± 0.0123	0.1574 ± 0.0124	< .0001
Area			0.5223
Northern	86 (48.86%)	148 (51.93%)	
Central and southern	90 (51.14%)	137 (48.07%)	
Age	48.8693 ± 11.7430	48.7895 ± 11.0194	0.9413
Exposure to SHS			0.9857
No	158 (89.77%)	256 (89.82%)	
Yes	18 (10.23%)	29 (10.18%)	
Exercise			0.4866
No	104 (59.09%)	159 (55.79%)	
Yes	72 (40.91%)	126 (44.21%)	
Drinking			0.0004
Never	160 (90.91%)	281 (98.60%)	
Former	4 (2.27%)	1 (0.35%)	
Current	12 (6.82%)	3 (1.05%)	
Body fat (%)	22.5494 ± 5.6312	31.1582 ± 6.2627	< .0001
BMI (kg/m^2^)	24.8437 ± 3.5941	23.0715 ± 3.3297	< .0001
Waist-hip ratio	0.8848 ± 0.0542	0.827 ± 0.0659	< .0001
Asthma			0.8177
No	167 (94.89%)	269 (94.39%)	
Yes	9 (5.11%)	16 (5.61%)	
Emphysema			0.3410
No	173 (98.30%)	276 (96.84%)	
Yes	3 (1.70%)	9 (3.16%)	

Continuous variables, e.g., age and BMI, are expressed as mean ± standard deviation (SD), while categorical variables, e.g., exercise and drinking, are expressed as percentages (%)

**Table 2:** Mean (± SD) concentrations of PM_2.5_ (μg/m^3^) from 2006 to 2011 in the northern and central/southern areas

Area	*n*	2006	2007	2008	2009	2010	2011
Northern	18	27.43 ± 5.03	28.69 ± 5.39	27.33 ± 5.48	25.96 ± 5.32	25.98 ± 5.37	26.61 ± 5.30
Central/southern	23	37.69 ± 3.59	38.03 ± 4.05	37.48 ± 4.59	38.44 ± 3.67	35.46 ± 3.14	36.75 ± 4.97

*SD* standard deviation, *n* number of monitoring stations

**Table 3:** Multiple linear regression showing beta coefficients of SOX2 promoter methylation in men

Variable	*β*	*P* value
Area (northern: reference)		
Central and southern	0.00331	0.0257
Age	0.00027	0.0002
Exposure to SHS (no: reference)		
Yes	0.00098	0.6719
Exercise (no: reference)		
Yes	− 0.00189	0.2019
Drinking (never: reference)		
Former	− 0.00361	0.4308
Current	0.00005	0.9853
Body fat (%)	− 0.00039	0.1280
BMI (kg/m^2^)	0.00056	0.1411
Waist-hip ratio	0.01619	0.3230
Asthma	− 0.00427	0.1835
Emphysema	0.00021	0.9687

**Table 4:** Multiple linear regression showing beta coefficients of SOX2 promoter methylation in women

Variable	*β*	*P* value
Area (northern: reference)		
Central and southern	0.00514	< .0001
Age	0.00028	< .0001
Exposure to SHS (no: reference)		
Yes	0.00161	0.3697
Exercise (no: reference)		
Yes	− 0.00100	0.3944
Drinking (never: reference)		
Former	− 0.00513	0.5870
Current	− 0.00261	0.6142
Body fat (%)	− 0.00019	0.4164
BMI (kg/m^2^)	0.00028	0.5265
Waist-hip ratio	− 0.01242	0.1891
Asthma	0.00005	0.9830
Emphysema	− 0.00025	0.9350

**Table 5:** Multiple linear regression showing the association between mean PM_2.5_ (μg/m^3^) from 2006 to 2011 and SOX2 promoter methylation

Variable	*β*	*P* value
PM_2.5_ (μg/m^3^)	0.00042	< .0001
Sex (women: reference)		
Men	0.00144	0.4793
Age	0.00028	< .0001
Exposure to SHS (no: reference)		
Yes	0.00142	0.3030
Exercise (no: reference)		
Yes	− 0.00145	0.1062
Drinking (never: reference)		
Former	− 0.00319	0.4290
Current	− 0.00074	0.7543
Body fat (%)	− 0.00030	0.0513
BMI (kg/m^2^)	0.00047	0.0854
Waist-hip ratio	− 0.00411	0.6063
Asthma	− 0.00153	0.4076
Emphysema	− 0.00073	0.7800

## Discussion

Much has not been done concerning the association between SOX2 promoter methylation and air pollution exposure. Apparently, the current study is the first to explore SOX2 promoter methylation in non-smoking Taiwanese adults based on exposure to air pollution. In both men and women living in the central and southern areas of Taiwan, SOX2 was significantly hypermethylated. The consistent hypermethylation in both sexes implies that SOX2 promoter methylation could be a potential biomarker for industrial air pollution exposure. It is always challenging to estimate individual exposure to air pollution due to the lack of validated tools. In most cases, individual exposure to air pollution is usually estimated using air quality indices from nearby monitoring stations [17]. Exposure misclassification might have been minimized in the current study as participants were stratified based on the degree of exposure to air pollution. The central and southern areas have a heavy concentration of industries compared to the northern areas [16, 19, 21]. The number 6 Naphtha Cracking Complex found in Central Taiwan is one of the biggest power plants in the world [22]. Moreover, the Formosa Petrochemical Corporation and many other industries are found in Central-Southern Taiwan [22]. These make the areas more prone to industrial emissions. Hence, they naturally serve as experimental fields for industrial air pollution. To minimize confounding due to smoking, only non-smokers were included in the final analysis and adjustments were made for exposure to SHS.

To combat smoking and its associated effects, e.g., non-communicable diseases, the Health Promotion Administration (HPA), Taiwan, aims to reduce smoking to 14% by 2025 compared to 20% in 2010 [23]. A decrease in smoking prevalence could subsequently lead to a decrease in smoking-related diseases. However, this does not eliminate the possibility of exposures to pollutants and their carcinogenic impacts. For example, NSCLC which is most common in non-smokers accounts for approximately 85% of lung cancer [7]. In addition, a greater percentage of known lung cancer patients in Asia are never-smokers [11, 13]. Furthermore, smoking-related female lung cancer cases in Taiwan are few [14].

DNA methylation might help in the molecular staging of cancer as precancerous and cancerous states might be identified through this epigenetic modification [24]. This might also help to explain the mechanism for the initiation and progression of some tumors. For instance, lung cancer has been associated with epigenetic silencing [24–26]. Promoter hypermethylation is believed to be the molecular mechanism of this epigenetic gene silencing [24]. In esophageal cancer, SOX2 promoter hypermethylation was thought to be the main mechanism behind the silencing of SOX2 gene [3]. Moreover, in esophageal and hypopharyngeal cancer, the absence of SOX2 expression was associated with poor prognosis [3, 27]. SOX2 silencing through hypermethylation might promote tumor initiation by evading cell-cycle arrest in addition to resisting apoptosis [3]. In the current study, SOX2 promoter hypermethylation was observed in individuals who resided in central and southern areas of Taiwan which have high levels of air pollution. Moreover, analysis of the KRAS gene promoter CpG sites also showed significant hypermethylation in participants residing in the central and southern compared with the northern areas. Exposure to air pollution predisposes to cancer [14–16]. SOX2 is believed to be a hallmark of lung cancer [2]. Therefore, in healthy non-smokers, SOX2 hypermethylation in air pollution areas might help to identify those at precancerous stages that have not been clinically diagnosed.

One of the limitations of this study is that the association between SOX2 promoter methylation and SOX2 gene expression was not determined. However, hypermethylation is believed to be associated with reduced gene expression [25]. Specifically, SOX2 promoter hypermethylation has been shown to be a vital epigenetic event that causes the silencing of the gene [3]. Moreover, it has been significantly associated with reduced expression of SOX2 RNA in choriocarcinoma and hydatidiform mole [28]. Another limitation of the study is that only non-cancerous individuals were included in this study. As such, the results may not reflect the methylation patterns in individuals with cancer.

## Conclusion

In conclusion, significant hypermethylation of the SOX2 promoter region was observed in both non-smoking men and women living in industrial areas with well-known higher levels of air pollution. SOX2 promoter methylation might serve as a biomarker for air pollution in industrial areas. Moreover, it might reflect predisposition to cancer. Hence, healthy non-smokers at precancerous stages who have not been clinically diagnosed could be identified and monitored. Subsequently, prompt actions could be taken against such a public health issue. It is recommended that more should be done to explore the relationship between SOX2 methylation and air pollution.

## Methods

### Data source

Data were retrieved from the Taiwan Biobank database (2008–2015). The Taiwan Biobank was established in 2005 with the aim of integrating the genetic and medical information of about 200,000 ethnic Taiwanese with no history of cancer [29, 30]. The enrollment of individuals in the Taiwan Biobank Project conforms to relevant regulations and guidelines. A total of 29 recruitment centers are distributed all over the country; each county or city possesses a minimum of one center [29]. A letter of consent was signed by each individual before data collection. Data were collected by well-trained researchers through questionnaires, physical examinations, and biochemical analyses of blood and urine samples.

### Data collection and study participants

Participants’ data on SOX2 promoter methylation, residence, age, smoking, exposure to SHS, exercise, drinking, body fat, BMI, WHR, asthma, and emphysema were extracted from the Taiwan Biobank dataset (2008–2015). Residence, age, smoking, exposure to SHS, exercise, drinking, asthma, and emphysema were self-reported while DNA methylation, body fat, BMI, and WHR were measured.

Venous blood (9 ml) was collected into sodium citrate tubes and transported to the lab under 4 °C. DNA was extracted from blood using Chemagic™ Prime™ instrument which is an automated chemical extraction machine that uses magnetized rods to separate nucleic acids from solutions. The DNA length was determined with the Fragment Analyzer (Agilent) while the purity was assessed using the optical density (OD) at 260/280. Samples with an OD 260/280 ratio of 1.6–2.0 were considered to be pure. Pure samples intended for long-term use were stored at − 80 °C. DNA samples were subjected to sodium bisulfite treatment using the EZ DNA Methylation Kit (Zymo Research, CA, USA). DNA methylation at each CpG site was determined with the Infinium® MethylationEPIC BeadChipEPIC array (Illumina Inc.) which covers 850,000 methylation sites. Details on this platform are described elsewhere [31–33]. The methylation levels were expressed as beta-values (*β*) which range between 0 and 1. *β*-values were determined using the formula *β = M*/(*M* + *U*), where *M* is the methylated intensity and *U* is the unmethylated intensity. Quality control was done according to the Illumina® GenomeStudio® Methylation Module v1.8 [34]. Samples with detection *P* value > 0.05 and bead counts < 3 were eliminated. Dye-bias across batches was adjusted by normalization, and background correction was performed. Outliers were removed using the median absolute deviation method.

Participants were considered to be from an area if they lived there for at least 3 months. Where participants lived was considered to be where they were likely to be exposed to air pollution. The residences were grouped into northern and central/southern regions. This is because PM_2.5_ pollution in the northern areas is lower compared to the central and southern areas [16, 18–20]. The northern areas included Taipei and New Taipei Cities, while the central/southern areas included Taichung and Nantou Cities, Changhua and Yunlin Counties, Chiayi County, Tainan, and Chiayi Cities. PM_2.5_ statistics in these areas were obtained from the Air Quality Monitoring Database (AQMD) set up by the Environmental Protection Administration, Taiwan. There were 41 air quality monitoring stations in the study areas. Of these stations, 18 were located in the northern region while 23 were located in the central/southern regions. Annual PM_2.5_ readings (2006–2011) from the various stations were used to determine the annual average concentration for each region.

Participants were considered as (1) non-smokers: never smoked or did not continuously smoke for 6 months or more; (2) former smokers: continuously smoked for at least 6 months but were currently not smoking; (3) current smokers: continuously smoked for 6 months or more and were currently smoking; (4) non-drinkers: no history of alcohol drinking or weekly drinking of less than 150 cc of alcohol for continuously 6 months; (5) former drinkers: abstained from drinking for over 6 months; (6) current drinkers: weekly drinking of at least 150 cc of alcohol continuously for 6 months; (7) physically active: exercised for > 150 min per week; and (8) exposed to SHS: exposure for at least 5 min per hour.

After excluding former and current smokers as well as those with incomplete information, a total of 461 non-smokers comprising 176 men and 285 women were included in our study. A total of 24 SOX2 CpG sites located on SOX2 promoter region were available in the Taiwan Biobank dataset. These sites were cg24513480, cg05664581, cg19258425, cg18148179, cg07747133, cg24782772, cg00666105, cg04948892, cg15106134, cg02573703, cg20106776, cg11129008, cg08062338, cg12930100, cg01023203, cg22530053, cg27331851, cg14783675, cg01340005, cg17051733, cg11142406, cg09530873, cg25933341, and cg08464053. The *β*-values at all the 24 SOX2 CpG sites were summed up, and the mean value was determined. In order to reach the conclusion that SOX2 is a reliable biomarker of cancer risk in air pollution areas, the mean *β*-value of CpG sites at the promoter of KRAS, another lung cancer risk gene, was determined. Ethical approval for this study was obtained from Chung Shan Medical University Institutional Review Board (CS2-17070).

### Statistical analysis

Data management and analyses were performed with the SAS 9.4 software (SAS Institute, Cary, NC). Continuous variables were analyzed using *t* test and expressed as mean ± standard deviation (SD) while categorical variables were analyzed using chi-square test and reported as percentages (%). Methylation data were normalized using the Illumina® GenomeStudio V2011.1 software [34]. The correction of cell-type heterogeneity was done with the R software using the Reference-Free Adjustment for Cell-Type composition (ReFACTor) method [35].

The association between SOX2 promoter methylation and residential area was determined using multiple linear regression. Multivariate adjustments were performed for age, exposure to SHS, exercise, drinking, body fat, BMI, WHR, asthma, emphysema, and cell-type composition. *P* values less than 0.05 were considered to be statistically significant.

## Additional file

Additional file 1: **Table S1**. Multiple linear regression showing beta coefficients of SOX2 methylation both in and outside the promoter region. Table S2. Multiple linear regression showing beta coefficients of SOX2 methylation in the exon. Table S3. Multiple linear regression showing beta coefficients of SOX2 methylation in the 3UTR region. Table S4. Multiple linear regression showing beta coefficients of KRAS promoter methylation. (DOCX 22 kb)

## Abbreviations

AC: Adenocarcinoma
BMI: Body mass index
CpG: Cytosine-phosphate-guanine
DNA: Deoxyribonucleic acid
HDL: High-density lipoprotein
LCC: Large cell carcinoma
LDL: Low-density lipoprotein
MOST: Ministry of Science and Technology
*n*: Sample size
NSCLC: Non-small cell lung cancer
SCC: Squamous cell carcinoma
SCLC: Small cell lung cancer
SD: Standard deviation
SHS: Second-hand smoke
SRY: Sex-determining region Y
TC: Total cholesterol
TG: Triglyceride
WHR: Waist-hip ratio
*β*: Regression coefficient
