# Supplementary material for: SOX2 promoter hypermethylation in non-smoking Taiwanese adults residing in air pollution areas
Source: Clin Epigenetics. 2019 Mar 12;11:46. doi: 10.1186/s13148-019-0647-8 (PMC6416982; doi:10.1186/s13148-019-0647-8)
Supplement: Supplementary file 1 — Table S1. Multiple linear regression showing beta coefficients of SOX2 methylation both in and outside the promoter region. Table S2. Multiple linear regression showing beta coefficients of SOX2 methylation in the exon. Table S3. Multiple linear regression showing beta coefficients of SOX2 methylation in the 3UTR region. Table S4. Multiple linear regression showing beta coefficients of KRAS promoter methylation. (DOCX 22 kb) [file 13148_2019_647_MOESM1_ESM.docx]

**Additional file 1: Table S1. Multiple linear regression showing beta coefficients of SOX2 methylation both in and outside the promoter region**

| **Variable** | **β** | **P-value** |
| --- | --- | --- |
| Area (Northern= reference) |  |  |
| Central and Southern | 0.00407 | <.0001 |
| Sex (Women= reference) |  |  |
| Men | 0.00378 | 0.0575 |
| Age | 0.00026 | <.0001 |
| Exposure to SHS (No= reference) |  |  |
| Yes | 0.00080 | 0.5508 |
| Exercise (No= reference) |  |  |
| Yes | -0.00143 | 0.1039 |
| Drinking (Never= reference) |  |  |
| Former | -0.00425 | 0.2802 |
| Current | -0.00084 | 0.7144 |
| Body fat (%) | -0.00022 | 0.143 |
| BMI (kg/m2) | 0.00022 | 0.4141 |
| Waist-hip ratio | -0.00331 | 0.6711 |
| Asthma (No= reference) |  |  |
| Yes | -0.00124 | 0.4933 |
| Emphysema (No= reference) |  |  |
| Yes | -0.00028 | 0.9120 |

**Table S2. Multiple linear regression showing beta coefficients of SOX2 methylation in the exon**

| **Variable** | **β** | **P-value** |
| --- | --- | --- |
| Area (Northern= reference) |  |  |
| Central and Southern | 0.0033 | 0.0233 |
| Sex (Women= reference) |  |  |
| Men | 0.01073 | 0.0014 |
| Age | 0.00020 | 0.0042 |
| Exposure to SHS (No= reference) |  |  |
| Yes | -0.00102 | 0.6512 |
| Exercise (No= reference) |  |  |
| Yes | -0.00099 | 0.5020 |
| Drinking (Never= reference) |  |  |
| Former | -0.00644 | 0.3295 |
| Current | -0.00104 | 0.7867 |
| Body fat (%) | 0.00002 | 0.9286 |
| BMI (kg/m2) | -0.00049 | 0.2765 |
| Waist-hip ratio | -0.00123 | 0.9247 |
| Asthma (No= reference) |  |  |
| Yes | 0.00003 | 0.9932 |
| Emphysema (No= reference) |  |  |
| Yes | 0.00118 | 0.7833 |

**Table S3. Multiple linear regression showing beta coefficients of SOX2 methylation in the 3UTR region**

| **Variable** | **β** | **P-value** |
| --- | --- | --- |
| Area (Northern= reference) |  |  |
| Central and Southern | 0.00467 | 0.0194 |
| Sex (Women= reference) |  |  |
| Men | -0.00951 | 0.0381 |
| Age | 0.00017 | 0.0745 |
| Exposure to SHS (No= reference) |  |  |
| Yes | 0.00399 | 0.1996 |
| Exercise (No= reference) |  |  |
| Yes | -0.00488 | 0.0162 |
| Drinking (Never= reference) |  |  |
| Former | -0.00826 | 0.3623 |
| Current | -0.00172 | 0.7454 |
| Body fat (%) | -0.00074 | 0.0328 |
| BMI (kg/m2) | 0.00123 | 0.0470 |
| Waist-hip ratio | -0.00344 | 0.8482 |
| Asthma (No= reference) |  |  |
| Yes | -0.00619 | 0.1369 |
| Emphysema (No= reference) |  |  |
| Yes | -0.00449 | 0.4455 |

**Table S4. Multiple linear regression showing beta coefficients of KRAS promoter** **methylation**

| **Variable** | **β** | **P-value** |
| --- | --- | --- |
| Area (Northern= reference) |  |  |
| Central and Southern | 0.02679 | 0.0009 |
| Sex (Women= reference) |  |  |
| Men | 0.04672 | 0.0121 |
| Age | 0.00110 | 0.0043 |
| Exposure to SHS (No= reference) |  |  |
| Yes | 0.01039 | 0.4067 |
| Exercise (No= reference) |  |  |
| Yes | 0.00264 | 0.7465 |
| Drinking (Never= reference) |  |  |
| Former | -0.01603 | 0.6611 |
| Current | 0.00882 | 0.6795 |
| Body fat (%) | 0.00096 | 0.4937 |
| BMI (kg/m2) | -0.00043 | 0.8617 |
| Waist-hip ratio | -0.24424 | 0.0008 |
| Asthma (No= reference) |  |  |
| Yes | 0.03063 | 0.0679 |
| Emphysema (No= reference) |  |  |
| Yes | 0.03634 | 0.1260 |
